# Impact of neoadjuvant therapy followed by laparoscopic radical gastrectomy with D2 lymph node dissection in Western population: A multi‐institutional propensity score‐matched study

**DOI:** 10.1002/jso.26657

**Published:** 2021-08-25

**Authors:** Umberto Bracale, Francesco Corcione, Giusto Pignata, Jacopo Andreuccetti, Pasquale Dolce, Luigi Boni, Elisa Cassinotti, Stefano Olmi, Matteo Uccelli, Monica Gualtierotti, Giovanni Ferrari, Paolo De Martini, Miloš Bjelović, Dragan Gunjić, Diego Cuccurullo, Antonio Sciuto, Felice Pirozzi, Roberto Peltrini

**Affiliations:** ^1^ Department of Advanced Biomedical Sciences University of Naples Federico II Naples Italy; ^2^ Department of Public Health University of Naples Federico II Naples Italy; ^3^ Department of General Surgery II Spedali Civili of Brescia Brescia Italy; ^4^ Department of General and Mininvasive surgery San Camillo Hospital Trento Italy; ^5^ Department of Surgery, Fondazione IRCCS Ca' Granda Ospedale Maggiore Policlinico, University Milano Italy; ^6^ Department of General and Oncologic Surgery San Marco Hospital GSD Zingonia Italy; ^7^ Department of Minimally Invasive Oncologic Surgery, Niguarda Hospital ASST Grande Ospedale Metropolitano Niguarda Milan Italy; ^8^ Department of Minimally Invasive Upper Digestive Surgery, Hospital for Digestive Surgery Clinical Center of Serbia Belgrade Serbia; ^9^ Department of General, Mini‐Invasive and Robotic Surgery Monaldi Hospital Naples Italy; ^10^ Department of General Surgery Santa Maria delle Grazie Hospital Pozzuoli Naples Italy

**Keywords:** gastric cancer, neoadjuvant therapy, laparoscopic gastrectomy, postoparative complications, oncological outcomes

## Abstract

**Background and Objectives:**

In the setting of a minimally invasive approach, we aimed to compare short and long‐term postoperative outcomes of patients treated with neoadjuvant therapy (NAT) + surgery or upfront surgery in Western population.

**Methods:**

All consecutive patients from six Italian and one Serbian center with locally advanced gastric cancer who had undergone laparoscopic gastrectomy with D2 lymph node dissection were selected between 2005 and 2019. After propensity score‐matching, postoperative morbidity and oncologic outcomes were investigated.

**Results:**

After matching, 97 patients were allocated in each cohort with a mean age of 69.4 and 70.5 years. The two groups showed no difference in operative details except for a higher conversion rate in the NAT group (*p* = 0.038). The overall postoperative complications rate significantly differed between NAT + surgery (38.1%) and US (21.6%) group (*p* = 0.019). NAT was found to be related to a higher risk of postoperative morbidity in patients older than 60 years old (*p* = 0.013) but not in patients younger (*p* = 0.620). Conversely, no difference in overall survival (*p* = 0.41) and disease‐free‐survival (*p* = 0.34) was found between groups.

**Conclusions:**

NAT appears to be related to a higher postoperative complication rate and equivalent oncological outcomes when compared with surgery alone. However, poor short‐term outcomes are more evident in patients over 60 years old receiving NAT.

## 1 INTRODUCTION

Gastric cancer is one of the most common cancers in the world^1^, ^2^ and it is the third primary cause of cancer‐related death around the world afflicting more than 400 000 patients each year in China^3^ and more than 10 000 cancer‐related deaths in the United States in 2017.^4^ According to Western guidelines patients with locally advanced gastric cancer (LAGC) should undergo radical gastrectomy with D2 lymph node dissection.^5^, ^6^ Additionally, neoadjuvant therapy (NAT) with a perioperative regimen is recommended for patients with more than or equal to Stage IB resectable gastric cancer.^7^ Benefits in terms of survival of this multimodal treatment are mainly supported by MAGIC^8^ and the FNCLCC‐FFCD^9^ trials that introduced NAT in the current clinical practice as a standard of care for LAGC in Western countries. However, some issues have arisen from these studies such as the inclusion of patients with lower esophagus or esophagogastric junction cancer in the analysis and an inadequate lymph node dissection in most cases. Over time, a perioperative regimen for treatment of LAGC was established as a procedural reference model within this setting.^10^, ^11^

Although it appears that NAT can be administered without increasing postoperative morbidity compared with gastrectomy alone, no definitive conclusions can be drawn^12^, ^13^ and furthermore, results from the CRITICS trial show that incomplete preoperative NAT due mainly to toxicity is an independent risk factor in developing postoperative complications.^14^ In Eastern Asia, upfront surgery (US) is still recommended as primary treatment and health insurance in Japan and South Korea does not support neoadjuvant treatment for surgically resectable LAGC,^10^ despite clinical evidence on the use of NAT are establishing in Japan.^15^, ^16^

Along with the controversial management of LAGC, minimally invasive surgery is yet another variable that needs to be evaluated. The adoption of a laparoscopic approach in the treatment of LAGC demonstrated beneficial short‐term outcomes and comparable long‐term outcomes over open surgery in multicenter Asian randomized controlled trials (RCTs).^17^, ^18^, ^19^, ^20^, ^21^ Laparoscopic gastrectomy (LG) is also gaining consensus in Europe^22^, ^23^ and United States^24^, ^25^ with satisfactory results in terms of oncological quality and postoperative morbidity. However, LG cannot be considered the gold standard for LAGC surgery and it remains limited to referral Centers with experience in the field.

As evidence on both NAT and LG have increased and they are recognized as promising treatment strategies in West, the role of NAT in patients exclusively undergoing laparoscopic radical gastrectomy with D2 lymph node dissection should be investigated.

The objective of this study is to compare short and long‐term outcomes of patients receiving NAT before surgery with those receiving surgery alone through a “real‐word” retrospective analysis from Western Centers with proven experience in laparoscopic gastric cancer surgery.

## 2 MATERIAL AND METHODS

### 2.1 Study population

A retrospective review of seven institutional databases was conducted to identify all patients who underwent LG for LAGC (Stages II and III) with curative intent from January 2005 to December 2019. Tumor stages followed the 8th edition of American Joint Committee on Cancer (AJCC) TNM staging system for gastric cancer.^26^ Patients who received neoadjuvant therapy (NAT group) were matched with patients who underwent upfront surgery (US group) according to the following criteria: age, gender, Body Mass Index (BMI), American Society Anesthesiologists (ASA) score, stage, surgical procedure, and lymphadenectomy. Data collection was approved by the Institutional Review Board of the leading hospital.

### 2.2 Surgical procedure

A preoperative workup was achieved by computed tomography (CT) scan of abdomen, thorax, and pelvis and endoscopy detecting precise tumor location. Biopsy confirmed cancer diagnosis in all cases. Endoscopic ultrasound (EUS) was used to provide further assessment of the T and N stage, when necessary. In selected cases, laparoscopy with peritoneal washings for malignant cells was performed to exclude occult metastatic disease involving peritoneum. A laparoscopic total gastrectomy was performed for pathologically confirmed cancers of the corpus/fundus of the stomach and a laparoscopic subtotal gastrectomy was performed for antral gastric tumors. A D2 lymph node dissection was then carried out according to the current clinical practice guidelines.^7^ All surgeries were performed by highly experienced surgeons. To ensure quality control of the surgical procedures, nonedited videos of both LTG and LSG performed by each participant were reviewed by the study coordinator before inclusion. The study coordinator has 30 years of experience in laparoscopic surgery. He performs more than forty gastrectomies per year for cancer, of which 70% with a minimally invasive approach.

### 2.3 Neoadjuvant chemotherapy

Neoadjuvant chemotherapy had been administered since 2011 in cases of cN + or ≥ cT2 tumor. Regimens consisted of a combination of Epirubicin–Cisplatin–5‐fluorouracil–Folinic Acid (ECF, 50 mg/m^2^ epirubicin, 60 mg/m^2^ cisplatin, and 5‐FU administered either by continuous infusion 200 mg/m^2^/d per 7 days via a CVC, administered every 3 weeks) or 5‐fluorouracil–Folinic Acid–Oxaliplatin–Docetaxel (FLOT, docetaxel (60 mg/m^2^), oxaliplatin (85 mg/m^2^), leucovorin (200 mg/m^2^), and 5‐fluorouracil (2600 mg/m^2^ as a 24 h infusion), all given on Day 1 and administered every 2 weeks.

### 2.4 Data collection and outcome measures

Baseline patient characteristics, intraoperative factors, and pathological tumor data were evaluated including gender, age, BMI, ASA status, tumor stage, type of surgery, operative time, blood loss, conversion rate, and intraoperative complications. Postoperative outcomes were evaluated in terms of complications and survival.

Postoperative complications occurred at any time during recovery or in the first 30 days after surgery were categorized based on Clavien–Dindo classification system.^27^ Anastomotic leakage was evaluated in accordance with the definition and grading system of the UK Surgical Infection Study Group.^28^ Postoperative mortality was defined as death by any cause within the first 30 or 90 days after surgery or at any time during a hospital stay.

Oncological outcomes in terms of disease‐free survival (DFS) and overall survival (OS) were reported for each group. Locoregional recurrence was defined as recurred carcinoma of the remnant gastric pouch or at anastomosis site or within the lymphatic drainage area of the region of the primary tumor, confirmed by CT scan and/or pathological examination. Distant metastases were defined as recurrent tumors in the peritoneum, liver, nonregional lymph nodes, or outside the abdominal cavity such as lung, bones, or other sites.

Follow‐up after surgery included physical examination every 3–6 months for the first 2 years and every 6–12 months for years 3–5. CT scan was performed every 6–12 months for the first 2 years, then annually for up to 5 years.

### 2.5 Statistical analysis

Preliminary analysis concerned the treatment of missing data. Very little data were missing for BMI (0.8% of the total) and time to first flatus (3% of the total), which were considered as missing at random. Missing value imputation was performed using the k nearest neighbors (KNN) algorithm,^29^ using the Euclidean distance as a distance metric in the multidimensional space.

Data were reported as a number of patients (%) for categorical variables and as mean ± standard deviation or median ± interquartile range (IQR), as appropriate, for quantitative variables.

A propensity score matching analysis with nearest‐neighbor matching was performed using the MatchIt R^30^ for optimal adjustment for potential confounding variables: age, sex, BMI, ASA, Stage Surgical procedure, and Lymphadenectomy. The matched samples, each one of 97 patients, were then compared in terms of operative details and postoperative outcomes using *χ* ^2^ test or Fisher's Exact test, as appropriate, for categorical variables, and Student's *t*‐test or Mann–Whitney test, as appropriate, for quantitative variables.

Logistic regression analysis was used to assess for predictors of postoperative complications in matched patients. Kaplan–Meier curves and log‐rank tests were used to compare overall survival and progression‐free survival between NAT + Surgery and Upfront Surgery group of patients.

For all statistical tests, a *p* value less than 0.05 was considered as statistical significance. All statistical analyses were performed using the R software for statistical computing.

## 3 RESULTS

The overall study population consisted of 366 patients. Before propensity score matching, statistically significant difference in stage tumor (*p* = 0.001) was recorded between NAT and US group (Table 1). After matching, 97 patients with a mean age of 70 years old remained for each group and the previous difference was eliminated. LSG was performed in 69.1% and 74.2% of cases in the NAT group and the US group, respectively (Table 2) whereas LTG was performed in the remaining cases (*p* = 0.524). D2 lymph node dissection was performed in almost all cases in both groups (97.9%). No statistically significant difference was found between the two groups concerning blood loss (*p* = 0.733), intraoperative complications (*p* = 0.434), operative time (*p* = 0.098), nodes harvested (*p* = 0.190), metastatic nodes (*p* = 0.400) except for the conversion rate that was higher in the NAT group than in the US group (23.7% vs. 11.3%; *p* = 0.038).

**Table 1:** Baseline patients' characteristics

	Before matching			After matching		
	NAT + Surgery *n* = 97 (%)	Upfront surgery *n* = 269 (%)	*p*	NAT + Surgery *n* = 97 (%)	Upfront surgery *n* = 97 (%)	*p*
Sex			0.290			1
M	59 (60.8)	145 (53.9)		59 (60.8)	60 (61.8)	
F	38 (39.1)	124 (46)		38 (39.1)	37 (38.1)	
Age (mean ± *SD*) years	69.4 ± 10.3	68.1 ± 12.1	0.334	69.4 ± 10.3	70.5 ± 11.8	0.501
BMI (mean ± *SD*) kg/m^2^	24.4 ± 4.2	24.9 ± 4.2	0.289	24.4 ± 4.2	25.0 ± 4.3	0.347
ASA			0.999			1
I–II	67 (69)	187 (69.5)		67 (69)	66 (68)	
III–IV	30 (30.9)	82 (30.4)		30 (30.9)	31 (31.9)	
Clinical Stage			0.001			1
II	24 (24.7)	149 (55.3)		24 (24.7)	24 (24.7)	
III	73 (75.2)	120 (44.6)		73 (75.2)	73 (75.2)	

Abbreviations: ASA, American Society Anesthesiologists score; BMI, body mass index; NAT, neoadjuvant therapy; *SD*, standard deviation.

**Table 2:** Operative details

(After matching)	NAT + Surgery *n* = 97 (%)	Upfront surgery *n* = 97 (%)	*p*
Surgical procedure *n* (%)			0.524
LSG	67 (69.1)	72 (74.2)	
LTG	30 (30.9)	25 (25.8)	
Up anastomotic reconstruction *n* (%):			0.637
Orringer	28 (28.9)	24 (24.7)	
OrVil	2 (2.1)	1 (1)	
Gastro Jejunal	67 (69.1)	72 (74.2)	
Lymphadenectomy			1
D2	95 (97.9)	95 (97.9)	
D2+	2 (2.1)	2 (2.1)	
Blood loss (median ± IQR)	75 ± 32.5	80 ± 32.5	0.733
Intraoperative complications	10 (10.3)	6 (6.2)	0.434
Conversion to open surgery	23 (23.7)	11 (11.3)	**0.038**
Operative time (mean ± *SD*)	254.7 ± 112.8	230.4 ± 89.2	0.098
Nodes harvested (median ± IQR)	23 ± 12	26 ± 14.5	0.190
Metastatic nodes (median ± IQR)	2 ± 5.5	2 ± 5	0.400

Abbreviations: IQR, interquartile range; LSG, laparoscopic subtotal gastrectomy; LTG, laparoscopic total gastrectomy; *SD*, standard deviation.

Postoperative clinical outcomes after matching are shown in Table 3. The overall complication rate was higher in the NAT group than the US group (38.1% vs. 21.6%; *p* = 0.019) but with no difference in terms of severity of complication (*p* = 0.110) or anastomotic (*p* = 0.999) and duodenal leakage (*p* = 0.497). Time to recovery of bowel function and length of hospital stay did not differ between groups and no deaths occurred in the first 30 days after surgery. At 90 days after surgery, the mortality rate was 0% and 2.1% in NAT and US groups, respectively.

**Table 3:** Postoperative outcomes

Postoperative outcomes (after matching)	NAT + Surgery *n* = 97 (%)	Upfront surgery *n* = 97 (%)	*p*
Overall postoperative complications	37 (38.1)	21 (21.6)	**0.019**
Anastomotic leak	2 (2.1)	3 (3.1)	0.999
Duodenal leak	0	2 (2.1)	0.497
Postoperative mortality	0	0	
Postoperative complications C–D:			0.110
I	60 (61.9)	76 (78.4)	
II	27 (27.8)	16 (16.5)	
III	4 (4.1)	2 (2.1)	
IIIb	5 (5.2)	3 (3.1)	
IV	0	0	
V	1 (1)	0	
Time to first flatus (median ± IQR)	3 ± 3	3 ± 2.5	0.169
Length of stay days (median ± IQR)	11 ± 6	10 ± 5.5	0.243
Recurrence	29 (29.9)	23 (23.7)	0.418
Time to recurrence (median ± IQR)	24 ± 19	26 ± 13.5	0.708
Death related to disease progression	29 (29.9)	23 (23.7)	0.418
Follow‐up (median ± IQR) months	26 ± 20	26 ± 12	0.893

Abbreviations: CD, Clavien–Dindo classification; IQR, interquartile range; NAT, neoadjuvant therapy.

**Figure 1:**
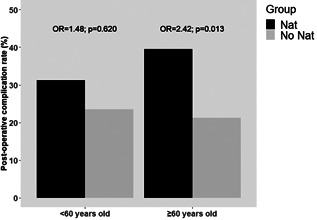
In older patients, postoperative morbidity rate was significantly higher in those who received neoadjuvant therapy. This association was not statistically significant in <60‐year‐old patients

Univariate logistic regression analysis was performed to further evaluate the risk factors for postoperative complications (Table 4). NAT was the only variable associated with complications (*p* = 0.013). Furthermore, a subgroup analysis was performed by dividing patients with complications into two groups: less than 60 years old and more than or equal to 60 years old. In older patients, postoperative morbidity rate was significantly higher in those who received NAT (odds ratio [OR] = 2.42, *p* = 0.013). This association was not statistically significant in less than 60‐year‐old patients (OR = 1.48, *p* = 0.620), see Figure 1.

**Table 4:** Logistic regression analysis of postoperative complications in propensity score‐matched patients

	Postoperative complications		Univariate analysis	
	Yes	No	OR	*p* value
	*n* = 58 (%)	*n* = 136 (%)		
Sex				
M	39 (67.2)	80 (58.8)	/	
F	19 (32.8)	56 (41.2)	0.70	0.271
Age	70.7 ± 12.1	69.6 ± 10.6	1.01	0.514
Age Cat.				
Age<60	9 (15.5)	24 (17.6)	/	0.718
Age≥60	49 (84.5)	112 (82.4)	1.17	
BMI	23.9 ± 3.6	25 ± 4.4	0.93	0.084
ASA:				
I–II	39 (67.2)	94 (69.1)	/	
III–IV	19 (32.8)	42 (30.9)	1.09	0.797
Operative time	255.1 ± 113.2	237.2 ± 96.9	1.01	0.266
Procedure:				
LSG	39 (67.2)	100 (73.5)	/	
LTG	19 (32.8)	36 (26.5)	1.35	0.375
Conversion	11 (19)	23 (16.9)	1.15	0.731
Blood loss	85 ± 42.5	75 ± 25	1.01	0.119
Intraop. Compl.	6 (10.3)	10 (7.4)	1.45	0.490
Harvested nodes	23 ± 14	25 ± 13	0.99	0.851
Metastatic nodes	2.5 ± 5.2	2 ± 5.8	1.06	0.121
Stage				
II	13 (22.4)	35 (25.7)	/	
III	45 (77.6)	101 (74.3)	1.20	0.624
Neoadjuvant therapy	37 (63.8)	60 (44.1)	2.23	**0.013**

Abbreviations: ASA, American Society Anesthesiologists score; BMI, body mass index; LSG, laparoscopic subtotal gastrectomy; LTG, laparoscopic total gastrectomy.

**Figure 2:**
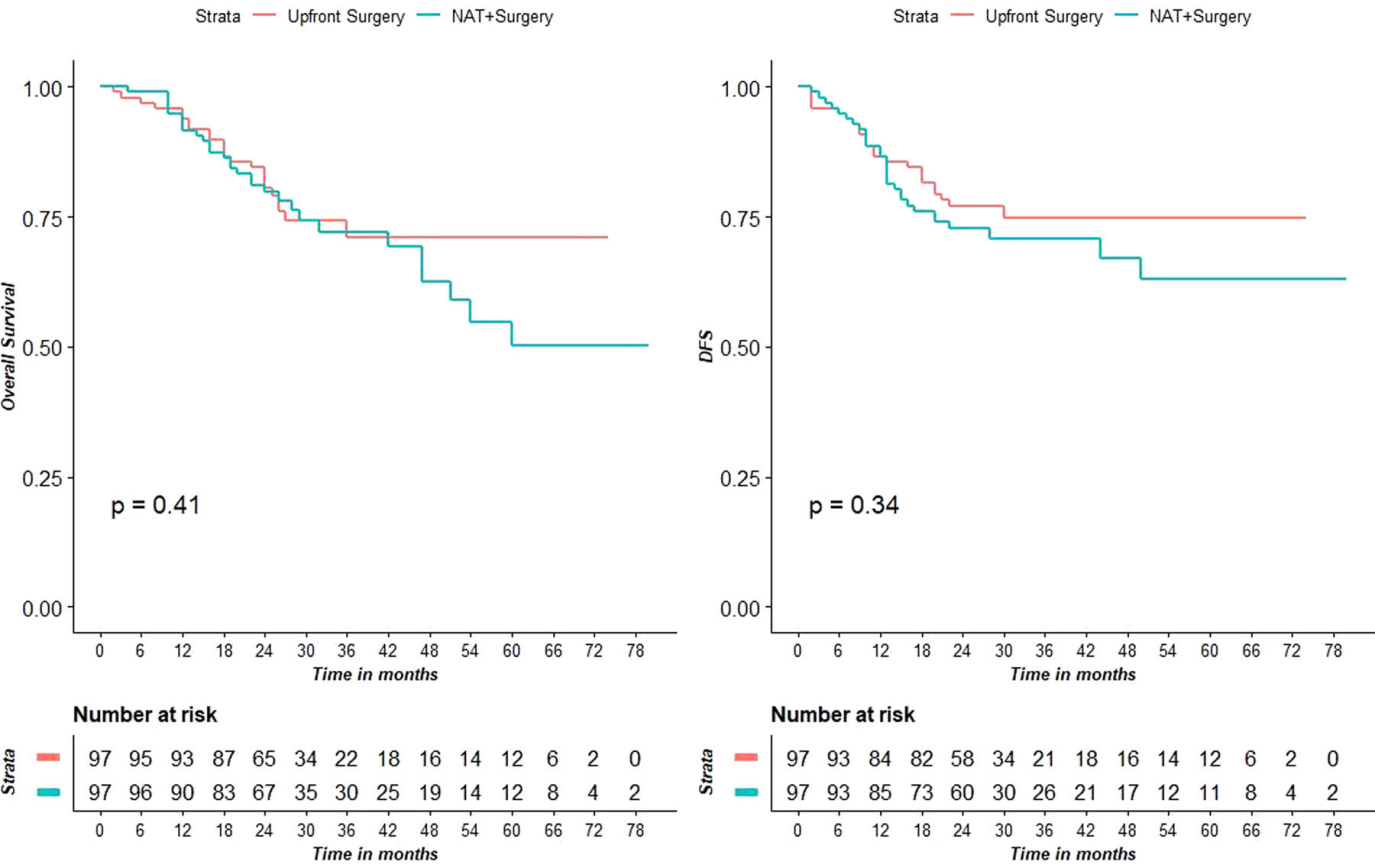
Kaplan–Meier curves showing the survival outcomes of the NAT + Surgery group and the US group and corresponding log‐rank *p* values after PSM. Overall survival (A). Disease‐free survival (B).

The recurrence rate was 29.9% in the NAT group and 23.7% in the US group (*p* = 0.418), with a median follow‐up of 26 months in each group. In the population of matched patients, 3‐years OS probability was 0.72 (95% confidence interval [CI] 0.62–0.83) in the NAT group and 0.71 (95% CI 0.61–0.83) in the US group, while 3‐years DFS was 0.71 (95% CI 0.62–0.81) in the NAT group and 0.75 (95% CI 0.66–0.85) in the US group. The difference in terms of OS and DFS between the two groups was not statistically significant (*p* = 0.41 and *p* = 0.34, respectively), see Figure 2.

## 4 DISCUSSION

The present study revealed higher conversion to open surgery and overall postoperative complications rates in patients with LAGC who had received NAT before LG compared with the US group. NAT is the only variable associated with the risk of postoperative morbidity before surgery, however, the statistical significance in morbidity rate disappears in patients under 60 years old with complications. Finally, no difference in overall survival and DFS was found between groups.

We reported outcomes of a “real‐world” retrospective analysis including patients with Stage II–III gastric cancer from seven institutions with a high level of experience in advanced laparoscopic surgery. We previously presented short and long‐term outcomes from the same series of over 300 patients who underwent LG.^31^ In the previous study, logistic binary regression for complications showed a statistically significant difference at univariate and multivariate analysis in patients who underwent NAT. Based upon these findings we decided to investigate the issue by way of a propensity score‐matched analysis to evaluate the role in NAT after LG with D2 lymph node dissection in a Western population.

After matching, we found that conversion to open surgery rate was higher in the NAT group compared with the US group (23.7% vs. 11.3%; *p* = 0.038) as well as the overall postoperative complication rate (38.1% vs. 21.6%; *p* = 0.019). In EORTC randomized trial 40954, Schuhmacher et al.^32^ compared the use of NAT before surgery with surgery alone with D2 lymph node dissection performed in over 90% of cases. This trial not only failed to demonstrate a survival benefit after NAT administration, but it also showed that postoperative complications were more frequent in the NAT group (27.1% vs. 16.2%; *p* = 0.09). However, the study included Stages III and IV adenocarcinoma of the stomach including Siewert I and II tumors of the esophagogastric junction differing from inclusion criteria of our study. The increase of surgical morbidity after NAT was also suggested by CRITICS trial.^14^ Patients who did not complete preoperative chemotherapy were more than twice as likely to develop postoperative complications (OR = 2.15, *p* = 0.003) and had a higher postoperative mortality rate (8.0%). Therefore, failure to complete NAT, mainly due to chemotherapy toxicity, was identified as risk factor for postoperative complications.

We hypothesize that several factors could affect outcomes after NAT as they relate to toxicity,^33^ a worse nutritional status, sarcopenia,^34^ and neutropenia.^35^ However, our findings differ from those of others western^36^, ^37^ and eastern^38^, ^39^, ^40^, ^41^ studies claiming that the administration of NAT is not associated with a greater risk of postoperative morbidity when compared to the United States. In the present study, two main aspects need to be considered foremost of which is a preoperative difference among population studies. Patients with a mean age of 70 years old were included in the present study and it is reasonable to assume that their outcomes would be different when compared to patients younger than 10–15 years^37^, ^38^, ^39^, ^40^ or 20 years.^41^ In fact, we demonstrated that the difference in complication rate between NAT and US group is relevant over 60 years old (*p* = 0.013) but not in younger patients (*p* = 0.620). Although it has been previously demonstrated that age is a significant predictor of postoperative complications,^42^, ^43^, ^44^ it is still controversial if NAT could negatively affect short‐term outcomes in older patients as reported by Fujitani et al.^45^ or not.^46^

A second aspect to consider is the minimally invasive approach for curative gastrectomy. In a comparative series of patients treated with gastrectomy and D2 lymphadenectomy, Wu et al.^38^ observed a more intraoperative blood loss over the surgery alone group (*p* < 0.04) because of NAT‐induced fibrosis surrounding lymph nodes. This could explain the higher rate of conversion to open surgery that we reported in the NAT group. However, only one international propensity score‐matched study compared the outcomes of patients who received LG with D2 lymphadenectomy with or without NAT.^47^ Although comparable postoperative complication rates were demonstrated between groups, age more than or equal to 60 was identified as a risk factor (OR 21.338; *p* < 0.001). Additionally, the remarkable difference in preoperative ASA score (ASA III = 2.3%) with the present study (ASA III–IV = 30%) could indirectly affect outcomes.

Based on this evidence, it can be assumed that age is not the only factor influencing postoperative morbidity, but also epidemiological data, anthropometric characteristics and therapeutic strategies which may vary may among geographic populations. In this setting, results of IMIGASTRIC trial^48^ confirmed that baseline patient selection and short‐term surgical outcomes of LG for gastric cancer are widely different between Eastern and Western surgical practices. Indeed, patient characteristics such as age, and BMI and the ASA score were higher in the West, as was the number of conversion to open surgeries, blood transfusions, and volume of estimated blood loss. No significant difference in complication rates was reported between groups but Grade III–IV complications occurred more frequently in the Western population. Finally, advanced age and a higher ASA score were independent risk factors for postoperative complications in the East, whereas NAT was an independent risk factor for postoperative morbidity, which is coherent with our findings.

No difference in OS (*p* = 0.41) and DFS (*p* = 0.34) was found between NAT and US groups in the present study. These findings seem apparently in contrast with the recommendations of the most important international guidelines^5^, ^7^ which recommended preoperative or perioperative chemotherapy as the standard of care in the case of LAGC. The objectives of NAT are to treat micrometastases and to improve R0 resection rate with D2 lymph node dissection. However, in Eastern Asia upfront surgery with adequate D2 lymphadenectomy is still preferred.

This evident contrast is probably due to the debated results of the MAGIC and the French FNCLCC/FFCD trials which confirmed better survival of patients who received perioperative chemotherapy compared with those treated with surgery alone. Indeed, some methodological drawbacks may affect results of these RCTs. In the MAGIC Trial,^8^ a greater proportion of stage T1–T2 cancer in the perioperative chemotherapy arm compared with the surgery alone arm was found and in more than 25% of patients the tumor location was lower esophagus or esophagogastric junction. Additionally, D2 lymphadenectomy was performed in only 40% of each group. Therefore, the survival benefit of NAT seems to be stronger for the esophagogastric junction and the lower esophagus location with respect to the stomach one.

Even in FNCLCC/FFCD trial,^9^ authors claim in the Results that chemotherapy effects were only significant in the esophagogastric junction subgroup, which included around two‐thirds of the patients because the two other subgroups (lower esophagus and stomach) were too small to distinguish between no effect and a small effect.

Conversely, our findings are consistent with more recent studies^36^, ^37^ demonstrating that NAT had not statistically significant effect on survival rates when compared with surgery alone in patients with LAGC and, based on these results, this appears to be confirmed even when a minimally invasive approach is adopted. Furthermore, even in the case of gastric signet ring cell carcinoma which is characterized by a worse prognosis, NAT does not appear to provide survival benefits compared with primary surgery.^49^, ^50^, ^51^ Therefore, with regard to the lack of difference in OS and DFS that we found between groups, long‐term outcomes seem absolutely justifiable after an adequate literature analysis, despite the need for well‐designed prospective trials to drawn definitive conclusions.

The current study has several limitations. In addition to its retrospective design, patients considered fit for surgery could more likely have been selected for the US group and we are aware that this cannot be overcome with propensity score‐matched analysis. Data used was collected from different institutions over a 15‐year period, and therefore the study suffers from a heterogeneity of the neoadjuvant regimens with no patients who received neoadjuvant therapy until 2010. Furthermore, the study provides neither the evaluation of NAT‐related toxicity nor a preoperative assessment of patients' conditions including morbidity and frailty index or performance status and sarcopenia evaluation.

## 5 CONCLUSIONS

This study suggests that NAT prior LG is related to a higher postoperative complication rate compared with surgery alone in patients with LAGC. The negative effect of NAT on postoperative morbidity is more evident in patients above 60 years old. NAT had a nonsignificant impact on DFS and OS when curative resection with D2 lymph node dissection was carried out. Further studies with more selective patient recruitment need to be conducted to define the real advantages of NAT before surgery in gastric cancer exclusively and to better understand the impact on postoperative and intraoperative complications in the patient population that undergoes laparoscopic gastrectomy with D2 dissection.

## CONFLICT OF INTERESTS

The authors declare that they are no conflict of interests.

## ETHICAL STANDARDS

The study was conducted according to the guidelines of the Declaration of Helsinki. Ethical review and approval were obtained by the Institutional Review Boards of the leading hospital.

## INFORMED CONSENT STATEMENT

All patients enrolled signed an informed consent for the surgical intervention and for all the procedure related to the treatment of the disease. This is an observational retrospective analysis focused on data of the common clinical practice.

## SYNOPSIS

A higher complication rate was observed after neoadjuvant therapy followed by laparoscopic gastrectomy compared to surgery alone. Difference in postoperative morbidity was significant in patients over 60 years of age. Comparable long‐term oncologic outcomes emerged between two groups.

## Data Availability

The data that support the findings of this study are available from the corresponding author upon reasonable request.
